# Surgical Challenges in Multi-Vessel Minimally Invasive Coronary Artery Bypass Grafting

**DOI:** 10.1155/2021/1195613

**Published:** 2021-12-29

**Authors:** Jia-Ji Liu, Qing-Yu Kong, Bin You, Lin Liang, Wei Xiao, Xiao-long Ma, Feng Pan, Li-Qun Chi

**Affiliations:** Department of Cardiac Surgery, Beijing Anzhen Hospital, Capital Medical University, Beijing Institute of Heart, Lung and Blood Vessel Diseases, Beijing, China

## Abstract

**Objectives:**

Minimally invasive coronary artery bypass grafting (MICS CABG) has emerged as an alternative treatment for patients with multi-vessel coronary artery disease, but there are certain surgical challenges inherent in the adoption of this approach. The present study was conducted to provide insight regarding the outcomes associated with our first 118 cases, to discuss the surgical difficulties encountered in these patients, and to outline the potential countermeasures.

**Methods:**

Between January 2017 and January 2020, 118 patients underwent multi-vessel MICS CABG. These patients were stratified into two groups based upon whether they did or did not experience surgical challenges, and early clinical outcomes were compared between these groups to assess the incidence of technical difficulties and associated factors.

**Results:**

Surgical challenges arose in 38 of the 118 cases in this study, including 13 cases of exposure-related difficulties, 11 cases of proximal anastomosis-related difficulties, 15 cases of distal anastomosis-related difficulties, 4 cases of LITA-related difficulties, and 3 cases of lung-related difficulties. Relative to the other 80 patients, those patients for whom intraoperative technical challenges arose experience significant increases in operative duration (4.94 ± 0.89 vs. 5.59 ± 1.11 h, *P*=0.001), intraoperative blood loss (667 ± 313 vs. 892 ± 532 mL, *P*=0.005), length of the ICU admission (17.59 ± 3.51 vs. 22.59 ± 17.31 h, *P*=0.015), and the duration of postoperative hospitalization (5.96 ± 1.23 vs. 6.71 ± 1.92 days, *P*=0.012). There were no significant differences between these groups with respect to the mean graft number, major complications such as stroke or organ dysfunction, or one-year graft patency.

**Conclusions:**

There is a substantial learning curve associated with performing off-pump MICS CABG to treat multi-vessel disease. Surgical challenges encountered during this procedure may increase the operative duration, intraoperative blood loss, ICU admission, and the duration of postoperative hospitalization. However, these issues do not appear to compromise the efficacy of complete revascularization, and early clinical outcomes associated with this procedure remain acceptable.

## 1. Introduction

The medical demand for minimally invasive procedures continues to grow, and there has been a concomitant rise in studies reporting on the feasibility of minimally invasive treatments for coronary artery disease (CAD) [1–4]. Minimally invasive coronary artery bypass grafting (MICS CABG) procedures employ a single small intercostal incision to reduce the invasiveness of this procedure while achieving an earlier return to full physical function. However, MICS CABG has rarely been adopted as a surgical approach for patients with multi-vessel lesions, likely owing to the surgical challenges and difficulties inherent in this procedure. While there have been studies discussing the surgical and long-term outcomes such as graft patency associated with the MICS CABG procedure, there has been little discussion in the literature pertaining to technical difficulties and management strategies associated with this procedure [5–8]. Given that the MICS CABG procedure is promising yet there are no fixed procedural steps, we hope that a discussion of our clinical experiences will help to guide the optimization of the quality and efficacy of this approach. Between January 2017 and January 2020, 118 instances of multi-vessel MICS CABG were performed via a single left intercostal incision at our facility in order to conduct complete revascularization. This study was thus formulated to discuss the clinical outcomes, surgical challenges, and factors associated with this procedure. In addition, perioperative outcomes were analyzed and potential countermeasures were explored.

## 2. Patients and Methods

### 2.1. Patient Population

All patients were treated at a single center between January 2017 and January 2020. All patients met the proper diagnostic indications for the off-pump coronary artery bypass grafting coronary, with coronary angiography (CAG) results revealing the damage to two or more vessels is defined by stenosis ≥50% in the left main artery and >75% in other target vessels. Preoperative cardiac ultrasonography results for these patients indicated a normal left ventricular systolic function (ejection fraction >45%) and a left ventricular end-diastolic diameter of <60 mm, and with no need for surgical treatment of the heart valves or large blood vessels, and no evidence of congenital heart structural diseases. Patients were excluded from this study if they required emergency surgery, suffered from any severe valve damage, ventricle aneurysms, or congenital heart diseases, exhibited severe liver or kidney dysfunction, suffered from diffuse coronary artery disease, presented with severe aortic calcification in chest computed tomography (CT) scans, or exhibited severe pleural adhesions, or a history of prior left thoracic surgery.

### 2.2. Surgical Techniques

**Figure 1:**
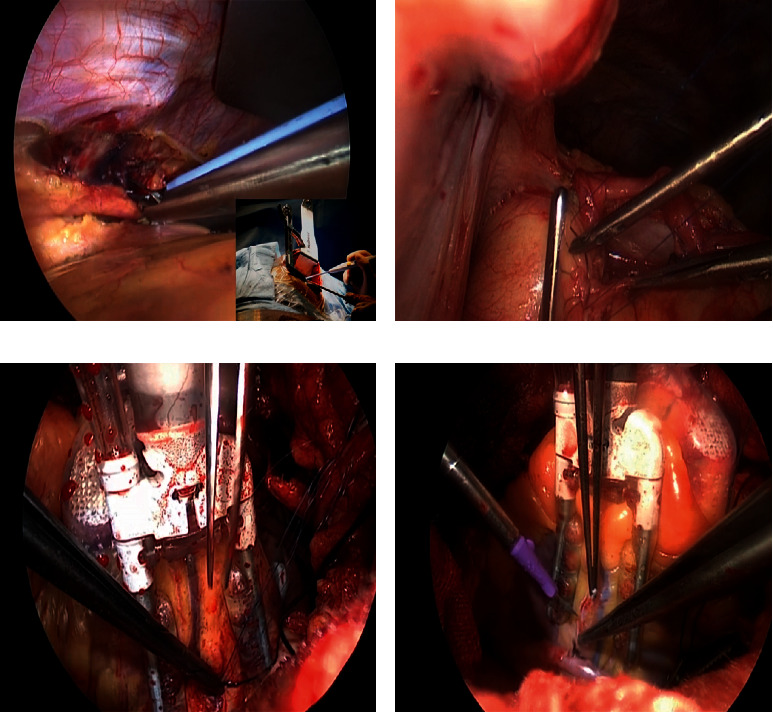
Surgical procedure. (a) LITA retractor (Thoratrak MIC S retractor system) was used to expose the chest wall space for LITA takedown. (b) Vein-aorta exposure and proximal anastomosis. (c) Vein to obtuse marginal branch anastomosis. (d) Vein to posterior descending artery anastomosis.

All procedures were performed under general anesthesia via double-lumen endotracheal intubation. Anesthetic techniques were largely the same as those employed for the off-pump coronary artery bypass grafting (OPCABG). In all cases, a double-lumen endotracheal tube was used to collapse the left lung to achieve the exposure of the left internal thoracic artery (LITA) for takedown and anastomosis. Padding was used to rotate the left scapula of each patient 30° to the right side, after which a left sub-mammary incision (8–12 cm long) was used to access the heart through the 4th or 5th intercostal space. The left lung was collapsed, and the positive end-expiratory pressure (PEEP) was set to 5 mmHg. A LITA retractor (Thoratrak MIC S retractor system) was used to expose the chest wall space (Figure 1(a)), and the LITA was harvested under direct vision from the 1^st^ to the 5^th^ intercostal space under the systemic heparinization (1 mg/kg). The anterior pericardium was incised in a longitudinal fashion, and the right pericardium near the ascending aorta was retracted and sufficiently suspended using three stitches up through the second intercostal space. Following the pericardial incision and suspension, the full-lung ventilation was restored, and the LITA-left anterior ascending artery (LAD) anastomosis were prioritized using a tissue stabilizer (TS2000, Medtronic Inc., Minneapolis, MN, USA) with a ValveGate™ hinged retractor to improve the hemodynamic stabilization. Next, proximal anastomosis was performed. The pericardium near the ascending aorta was retracted sufficiently, the aorta-pulmonary artery interval was freed, and a 4 × 4 cm gauge was placed at the right side of the ascending aorta. A side vascular clamp (Cardio Medical GmbH, Langenhagen, Germany) was then placed on the ascending aorta for proximal anastomosis using the ValveGate™ PRO needle holder, ring tissue forceps, and a knot pusher (Figure 1(b)). Other distal anastomoses (Figures 1(c) and 1(d)) can be completed as in the conventional OPCABG procedure using both tissue and *Octopus* stabilizers (29800 Medtronic Inc., Minneapolis, MN, USA). The transit-time flow measurement (TTFM) was employed to assess the intraoperative blood flow after vascular anastomosis using a QuickFit TTFM probe (Medistim VeriQ, Oslo, Norway). A single drainage tube was placed in the left thoracic cavity through the 6th intercostal space before closure.

### 2.3. Surgical Challenges

Surgical challenges were defined as any unexpected or specific obstacles that were associated with the minimally invasive access approach or the customized equipment used to conduct this procedure that might be encountered by a surgeon experienced in the OPCABG procedure. Surgical challenges encountered by our team when performing the MICS CABG procedure are summarized in Table 1 and include issues about exposure, proximal and distal anastomosis, LITA availability, and lung problems. Patients that exhibited surgical challenges were assigned to group A, while all other patients were assigned to group B.

### 2.4. One-Year Follow-Up Coronary Computed Tomography Angiography

Postoperative one-year coronary computed tomography angiography (CTA) was performed to assess the short-term graft patency. Fitzgibbon scores were used to define the grades and patency for both LIMA and vein grafts [9]. An unimpaired graft was given a patency grade of “A”, while a graft with impairment but <50% stenosis was given a grade of “B”, and an occluded or suspiciously underdeveloped graft was given a grade of “O”.

### 2.5. Statistical Analysis

Continuous variables are given as means with standard deviations (SDs), while categorical variables are given as frequencies or percentages. Data were analyzed using SPSS statistics v 22.0 (SPSS, Inc., IBM, Chicago, IL). Two-tailed *t*-tests, Chi-squared tests, or Fisher's exact test were used to compare the patient characteristics and clinical challenges between groups, as appropriate. A *P* value <0.05 was considered significant.

## 3. Results

Between January 2017 and January 2020, 118 patients underwent MICS CABG in our center. Procedural characteristics and certain postoperative outcomes for these patients are listed in Table 2. The mean number of distal anastomoses was 3.19 ± 0.75, with 23, 50, 44, and one patient undergoing two-, three-, four-, and five-vessel graft procedures, respectively. In 114 patients, the LITA was used for grafting. In one patient, the LITA and right internal thoracic artery (RITA) were both used via the same operative route. The distal anastomosis procedures covered all regular zones of culprit lesions in these patients. The average operative duration, including anesthesia, was 5.15 ± 1.01 h for the overall patient cohort. An intra-aortic balloon pump (IABP) was used for one patient during the surgery after ventricular fibrillation turned to sinus rhythm. However, no patients required conversion to sternotomy or cardiopulmonary bypass (CPB). One patient underwent a repeat thoracotomy due to bleeding. No major perioperative complications such as stroke or organ dysfunction were detected for patients in this case series.

Surgical challenges encountered by our team when performing the MICS CABG procedure are summarized in Table 1 and include issues pertaining to exposure, proximal and distal anastomosis, LITA availability, and lung problems. In total, 38 cases (32%) in this case series presented with such technical challenges, and the majority of these cases were earlier in the series. The TTFM was used as a routine approach to measure the instantaneous graft patency as surgeons became more familiar with the procedure owing to its unfamiliar anastomosis depth and angle. In cases where unsatisfactory LAD anastomosis results were detected, distal vein-patch angioplasty was employed in 2 cases, while an additional vein to distal LAD anastomosis was performed in 3 cases, and endarterectomy and reanastomosis were performed in 2 cases. To address three cases with right coronary artery (RCA) exposure challenges, a combination of techniques including *Octopus* stabilizers and pericardial retraction was employed. Patients that exhibited surgical challenges were assigned to group A, while all other patients were assigned to group B. There were no differences in baseline demographics between these groups except for gender ratio (Table 3). Surgical details and clinical outcomes for these two patient groups are summarized in Table 4. Relative to patients in group A, those in group B exhibited significantly decreased operative duration (4.94 ± 0.89 vs. 5.59 ± 1.11 h, *P*=0.001), intraoperative blood loss (667 ± 313 vs. 892 ± 532 mL, *P*=0.005), length of intensive care unit (ICU) admission (17.59 ± 3.51 vs. 22.59 ± 17.31 h, *P*=0.015), and duration of postoperative hospital stays (5.96 ± 1.23 vs. 6.71 ± 1.92 days, *P*=0.012). Patients in group B also exhibited benefits in 24 h drainage, drainage removal time, and the incidence of pleural effusion relative to patients in group A. There were no significant differences in mean grafts or in the incidence of major postoperative complications such as stroke or organ dysfunction when comparing these groups. In group A, the IABP and repeat thoracotomy were employed for 1 patient each, as discussed above, but no patient in either group required conversion to sternotomy or the use of CPB. There were 3 cases in which the MICS CABG was combined with other procedures. In one case, chest CT scans revealed an upper left lung mass that was confirmed as a tuberculoma following concomitant resection, while the other two cases entailed thymoma treatment.

Over the one-year follow-up period, 104 patients underwent CTA (88.14%), with 335 grafts being available for short-term clinical assessment. The overall graft patency proportion for patients in group A and group B were 91.82% and 94.67% (*P*=0.312), respectively, with respective LITA graft patency proportion of 96.88% and 97.06% (*P*=0.960), and vein graft patency proportion of 88.46% and 93.63% (*P*=0.171). Thus. there were no significant differences in the short-term graft patency between these groups (Table 5).

## 4. Discussion

Over the past decade, there has been a growing demand for minimally invasive cardiac surgical techniques [1–4]. While there have been a series of studies exploring surgical outcomes and angiographic graft patency associated with the MICS CABG procedure, insufficient attention has been paid to the technical challenges associated with this procedure and the management thereof [5–8]. In general, to achieve a good result in minimally invasive surgery, cardiac surgeons need to overcome a substantial learning curve [10]. The MICS CABG was initiated in 2005 with the aid of femoral cardiopulmonary bypass, during which, the major surgical challenge was the feasibility of handsewn proximal graft anastomoses onto the ascending aorta [2, 11]. To achieve optimal efficacy of the off-pump MICS CABG, the surgical team needs to master the methods of patient positioning, lung ventilation, the setup of an epicardial stabilizer/apical positioner and get familiar with distant aortic control and side-clamping and exposure of target vessels via a small thoracotomy [12]. The present study was conducted to detail the types and incidence rates of technical challenges encountered by our surgeons performing MICS CABG operations and to discuss relevant factors associated with such challenges. Short-term clinical outcomes were assessed, and appropriate countermeasures were outlined. As the MICS CABG approach continues to evolve through technical and technological improvements, we hope that our experience will guide efforts to improve the quality and uptake of this promising minimally invasive means to treating patients with multi-vessel disease.

The mean number of grafts per patient in our 118-case series was 3.19 ± 0.75, including the LAD, diagonal branch, obtuse marginal branch, the posterior branch of the left ventricle, posterior descending branch, marginal branch, and RCA grafts. Importantly, this MICS CABG approach was able to achieve complete revascularization across this broad range of graft types. In this case series, female patients tended to present with more technical challenges, likely due to the thinner grafts, thinner target vessels, and smaller chest cavities present in these patients, all of which can enhance the operative difficulty. Patients in the group that exhibited technical challenges exhibited increases in operative duration, length of ICU admission, duration of postoperative hospitalization, drainage during the first 24 h, and intraoperative blood loss. Importantly, however, there were no significant differences in mean grafts between groups or in the incidence of major postoperative complications including stroke, organ dysfunction, or mortality. No conversion to sternotomy or the CPB use was evident in either group. There were also no significant differences in short-term graft patency when comparing these two groups. Overall, technical challenges were encountered in 32% of the cases in our series. While this rate may seem high, all challenges were successfully addressed and appropriate countermeasures were implemented, and the clinical outcomes of these obstacles thus seem acceptable. These procedures were performed by surgeons proficient in performing the traditional OPCABG operations, and challenges were likely attributable to unexpected or specific difficulties associated with the adaptation to the unfamiliar surgical equipment or surgical field used for the MICS CABG procedure. There is an inherent learning curve for surgeons conducting multi-vessel lesions off-pump MICS CABG [11–13]. Primary technical challenges encountered in our case series were associated with issues of exposure, proximal and distal anastomosis, LITA availability, and lung problems. Below, we discuss approaches to addressing such issues that arise in the context of MICS CABG.

### 4.1. Exposure Problems and Limited Access

Exposure problems associated with limited access were observed in 9 cases in the present series. Adhesions of either the pleura or pericardium were carefully ablated, and one patient presented with pectus carinatum. This surgical route is relatively unfamiliar to cardiac surgeons, and consideration of the pertinent anatomy is very important in order to minimize the risk of damaging the lung tissue or the phrenic nerve. Selecting an appropriate intercostal route can be beneficial. Higher intercostal access routes can impair exposure, particularly when performing right coronary artery system anastomosis, while lower access routes can create difficulty in the context of proximal anastomosis. In 85% of our cases, we chose the 5^th^ intercostal space as an access route. One of the main advantages of the MICS CABG procedure is that it avoids complications associated with sternotomy, and the additional expansion of the intercostal incision or expansion to the adjacent intercostal space under the same skin incision are both viable alternatives that can be implemented without impacting the surrounding bones.

### 4.2. Proximal Anastomosis

When conducting the preoperative evaluation of MICS CABG patients, chest X-rays and chest CT scans must be performed given that surgeons cannot accurately feel the degree of aortic plaque or calcification by hand through a 6–10 cm incision. In addition, surgeons may not be able to detect comorbid diseases. For example, one patient in this series exhibited an upper left lung mass (2 × 3 cm) that was detected during CT scanning and that was later found to be a tuberculoma upon pathological evaluation following the concomitant resection. In a two-vessel lesion patient with severe calcification of the aorta, bilateral internal thoracic artery in situ was performed in place of vein grafting. Proximal anastomosis can be challenging owing to the depth and angle at which the procedure is performed, necessitating the use of a long needle holder, ring tissue forceps, and a knot pusher to access the site. Anastomosis-associated bleeding was evident in 6 cases in the present series, with reclamping and the placement of additional stitches ultimately being required for these patients. Vein to aorta anastomosis is the key technique when conducting MICS CABG procedures, as it allows surgeons to achieve complete anatomical grafting much as in the conventional OPCABG procedure. Some studies have reported the left posteroinferiorly displacement of the right ventricular outflow tract with an epicardial stabilizer as a means of creating space for proximal anastomosis, but this strategy has the potential, may cause transient pulmonary hypertension, potentially leading to subsequent right heart injury [14–16]. In our experience, sufficient retraction of the pericardium near the ascending aorta (normally three stitches), freeing of aorta-pulmonary artery interval, and gauge placement at the right side of the aorta are all effective approaches to anastomosis site exposure. Vascular clamping can further retract the ascending aorta. This method can achieve sufficient exposure with a pulmonary no-touch technique.

### 4.3. Distal Anastomosis

The TTFM is a routine approach to assess instantaneous graft patency. We routinely prioritize the LIMA-LAD anastomosis as a means of achieving early LAD revascularization in order to improve the safety of subsequent procedures, after which we measure the LITA-LAD TTFM. There were 8 cases in this series in which the LIMA-LAD TTFM results were unsatisfactory (velocity <20 mL/min, PI > 3). In 2 cases, we extended the LAD arteriotomy distally several millimeters to overstep the plaque, after which the LITA was reanastomosed on a long vein segment. In 2 cases, an endarterectomy was performed. In the other cases, we grafted an additional vein proximally with the saphenous vein graft to the distal LAD, thereby preparing a Y-shaped graft. In these cases, the final LAD TTFM results were much improved. All cases in this series were performed without pump assist. The distal anastomosis exposure instead relied upon pericardial retraction and the use of a tissue stabilizer. In some cases, an *Octopus* stabilizer can improve the exposure of deeper sites such as the RCA main branch. We experienced 3 cases of RCA endarterectomy and anastomosis through the 5^th^ intercostal space. Overall, these findings demonstrate that the off-pump MICS CABG is a feasible approach to achieve complete revascularization feasible for patients with multi-vessel CAD.

### 4.4. LITA Unavailability

Four patients in this series exhibited LITA unavailability, which occurred due to damage in one case, and due to a vascular ultrasound diameter <0.15 cm and velocity <45 cm/s, in which case the grafts were discarded by choice. In these four cases, Y-shaped all vein grafts were instead employed and were prepared via Y-shaped end-to-side vein grafting before proximal and distal anastomosis.

### 4.5. Intolerance for Single-Lung Ventilation

The use of a double-lumen endotracheal tube is standard for MICS CABG procedures. However, there were times when our anesthetists clamped the inlet supply tube of the left lung that patients were found to be unable to single-lung ventilation. Sixt et al. reported on the use of a straining pericardium method in which five to eight pledgeted pericardial sutures were employed to achieve continuous full-lung ventilation during the MICS CABG [17]. This technique necessitates the use of more incision sites to externalize these pericardial sutures. The single-lung ventilation provides the most benefit during the LITA harvesting and proximal anastomosis. During the remainder of the procedure, we believe that regular pericardial retraction should provide adequate operative space. As such, in three cases in our series, we implemented intermittent full-lung ventilation, decreased tidal volume, an increased respiratory rate, and placed a wet gauze to press the lung tissue in order to maintain the oxygen saturation at 90% while providing appropriate surgical exposure.

In this study, we have clinical outlines associated with our first 118 multi-vessel MICS CABG patients and discussed related surgical difficulties and appropriate countermeasures. There is a significant learning curve for surgeons learning to perform this multi-vessel lesion off-pump MICS CABG procedure, for experienced cardiac surgeons this curve primarily involves adaptation to the appropriate surgical route and equipment required for this procedure. Proximal anastomosis is often the cause of procedure-related obstacles, although the degree of complexity is always dependent on the damage to the target vessel. The incidence of technical challenges can increase the operative duration, intraoperative blood loss, duration of ICU admission, length of postoperative hospitalization, and first 24 hour drainage. However, even in these cases, complete revascularization does not appear to be compromised, and both postoperative complications and short-term graft patency are acceptable in treated patients. Appropriate quality control measures and further technique refinement are indispensable for this procedure.

### 4.6. Limitations

There are certain limitations to this study. For one, this was a retrospect, single-center, observational study. Second, coronary CTA was used to establish postoperative follow-up outcomes, even though coronary CTA exhibits only a moderate degree of diagnostic specificity and positive predictive value, and has a tendency to overestimate the degree of stenosis as in the presence of anastomotic lesions. Third, the MICS CABG procedure is associated with a learning curve that likely varies based upon the experience of the surgical team. Despite these limitations, we believe that this study serves as a valuable overview of some of the common challenges that may be encountered by cardiac surgeons and appropriate practical countermeasures.

## Figures and Tables

**Table 1:** Surgical challenges and clinical incidence (*n* = 118).

	Number	(%) (*n*/118)
Total number of cases (*n*, mean ± SD)	38	32
Exposure problems of access		
Pleural adhesion	7	6
Pericardium adhesion	1	2
Chest deformity	1	2
Expansion of incision	4	3
Proximal anastomosis		
Severe calcification of aorta (LITA + RITA)	1	2
Anastomosis bleeding and reclamping	6	5
Conversion to adjacent intercostal space	4	3
Unsatisfied TTFM results of LAD anastomosis		
Endarterectomy or distal patch angioplasty	4	3
Another vein-LAD graft	3	3
Other distal anastomoses		
Diffused plague and occlusion	4	3
Endarterectomy	3	3
Undisposed	1	2
RCA exposure	3	3
LITA unavailability	4	3
Y-shaped all vein grafts	4	3
Intolerance of single-lung ventilation	3	3

LITA, left internal thoracic artery. RITA, right internal thoracic artery. TTFM, transit time flow measure. LAD, left anterior descending artery. RCA, right coronary artery.

**Table 2:** Procedural characteristics and postoperative outcomes (*n* = 118).

Characteristics	MICS (*n* = 118)
Mean number of grafts per patient (*n*, mean ± SD)	3.19 ± 0.75
Five-vessel grafts, *n* (%)	1 (0.85)
Four-vessel grafts, *n* (%)	44 (37.29)
Three-vessel grafts, *n* (%)	50 (42.37)
Two-vessel grafts, *n* (%)	23 (19.49)
LITA, *n* (%)	114 (96.61)
RITA, *n* (%)	1 (0.85)
Distal anastomoses	
Left anterior descending artery, *n* (%)	115 (97.46)
Diagonal branch, *n* (%)	58 (49.15)
Ramus branch, *n* (%)	8 (6.78)
Obtuse marginal branch, *n* (%)	94 (79.66)
Posterior descending artery, *n* (%)	65 (55.08)
Posterior branch of the left ventricle, *n* (%)	14 (11.86)
Right coronary artery, *n* (%)	9 (7.63)
Operation duration (hour, mean ± SD)	5.15 ± 1.01
Intraoperative blood loss (ml, mean ± SD)	739.41 ± 408.71
Perioperative mortality, *n* (%)	0 (0)
Intra-aortic balloon pump, *n* (%)	1 (0.8)
Conversion to sternotomy or CPB, *n* (%)	0 (0)
Repeat thoracotomy due to bleeding, *n* (%)	1 (0.8)

LITA, left internal thoracic artery. RITA, right internal thoracic artery. CPB, cardiopulmonary bypass.

**Table 3:** Patients' profiles with (group A) and without surgical challenges (group B).

	Group A *n* = 38	Group B *n* = 80	*P* value
Female, *n* (%)	7 (18.4)	3 (3.8)	0.007
Age (y, mean ± SD)	62.82 ± 10.00	60.01 ± 8.30	0.112
Height (cm, mean ± SD)	168.92 ± 6.22	171.08 ± 6.27	0.083
Body weight (kg, mean ± SD)	73.16 ± 9.85	72.53 ± 10.24	0.751
BMI (kg/m^2^, mean ± SD)	25.74 ± 3.10	24.51 ± 3.95	0.095
Ejection fraction (%, mean ± SD)	61.76 ± 6.32	61.01 ± 5.51	0.533
LVEDD (mm, mean ± SD)	47.76 ± 4.00	48.69 ± 4.72	0.299
Diameter of LITA (cm, mean ± SD)	0.21 ± 0.03	0.21 ± 0.26	0.997
Velocity of LITA (cm/s, mean ± SD)	77.63 ± 21.25	75.05 ± 19.57	0.516
Smoking history, *n* (%)	14 (36.8)	39 (48.8)	0.224
COPD, *n* (%)	3 (7.9)	8 (10.0)	0.713
CT results of any brain infarction, *n* (%)	12 (31.6)	22 (27.5)	0.648
Diabetes mellitus, *n* (%)	16 (40.0)	33 (41.2)	0.930
Hypertension, *n* (%)	25 (65.8)	41 (41.3)	0.137

BMI, body mass index. LVEDD, left ventricular end diastolic diameter. LITA, left internal thoracic artery. COPD, chronic obstructive pulmonary disease.

**Table 4:** Surgical information and clinical outcomes.

	Group a *n* = 38	Group B *n* = 80	*P* value
Grafts number (*n*, mean ± SD)	3.26 ± 0.76	3.16 ± 0.75	0.500
Operation duration (hour, mean ± SD)	5.59 ± 1.11	4.94 ± 0.89	0.001
Combined operations (*n*)	3 (7.9)	0 (0)	—
Intraoperative blood loss (ml, mean ± SD)	892 ± 532	667 ± 313	0.005
Intraoperative blood transfusion rate, *n* (%)	3 (7.9)	0 (0)	0.011
30-day mortality, *n* (%)	0 (0)	0 (0)	1.000
New onset stroke, *n* (%)	0 (0)	0 (0)	1.000
Atrial fibrillation, *n* (%)	6 (16.2)	6 (7.5)	0.148
First 24-hour drainage (ml, mean ± SD)	479 ± 343	337 ± 184	0.004
Chest drainage removal time (day, mean ± SD)	3.37 ± 0.91	2.98 ± 0.66	0.009
Pleural effusion, *n* (%)	7 (18.4)	2 (2.5)	0.002
Postoperative ventilation duration (hour, mean ± SD)	18.31 ± 13.71	15.14 ± 3.83	0.057
Pulmonary atelectasis or pneumothorax	4 (10.5)	1 (1.3)	0.019
Length of ICU stays (hour, mean ± SD)	22.59 ± 17.31	17.59 ± 3.51	0.015
Postoperative hospital stays (day, mean ± SD)	6.71 ± 1.92	5.96 ± 1.23	0.012
IABP, *n* (%)	1 (2.6)	0 (0)	0.145
ECMO, *n* (%)	0 (0)	0 (0)	1.000
CRRT, *n* (%)	0 (0)	0 (0)	1.000
Conversion to sternotomy or CPB, *n* (%)	0 (0)	0 (0)	1.000
Repeat thoracotomy due to bleeding, *n* (%)	1 (2.6)	0 (0)	0.145

IABP, intra-aortic balloon pump. ECMO, extracorporeal membrane oxygenation. CRRT, continuous renal replacement therapy. CPB, cardiopulmonary bypass.

**Table 5:** One-year follow-up of CTA.

Characteristics	Group A *n* = 38	Group B *n* = 80	*P* value
Patients with follow-up CTA	33	71	—
Total grafts number	110	225	—
LITA grafts	32	68	—
SVG grafts	78	154	—
Fitzgibbon grade A	95	201	—
Fitzgibbon grade B	6	12	—
Fitzgibbon grade O	9	12	—
Total graft patency (%)	91.82	94.67	0.312
LITA patency (%)	96.88	97.06	0.960
SVG patency (%)	88.46	93.63	0.171

CTA, computed tomography angiography. SVG, saphenous vein graft.

## Data Availability

The data used to support the findings of this study are available from the corresponding author upon request.
